# 227. *Staphylococcus aureus* bacteremia: back to normal

**DOI:** 10.1093/ofid/ofad500.300

**Published:** 2023-11-27

**Authors:** Miguel Sebastian Pedromingo Kus, Ana Cristina Antolí, Nuria Iglesias Núñez

**Affiliations:** Hospital Nuestra Señora de Sonsoles, Ávila, Castilla y Leon, Spain; Hospital Nuestra Señora de Sonsoles, Ávila, Castilla y Leon, Spain; Hospital Nuestra Señora de Sonsoles, Ávila, Castilla y Leon, Spain

## Abstract

**Background:**

*Staphylococcus aureus* bacteremia (SAB) source is often unclear. One of the most common origins of SAB is cutaneous wounds and intravascular catheter-related bloodstream infection (CRBSI). Moreover, as our study group previously described, abscess formation and SAB following intramuscular injections are rare but possible complications from vaccines/inoculations. After an initial period of heightened SAB due to COVID-19 vaccines, we noticed a reversal of this circumstance.

**Methods:**

In this case series, we present a cohort of 100 patients with SAB from January 1, 2021 through April 24, 2023, attended in our Institution (Hospital Nuestra Señora de Sonsoles, Ávila, Spain). We identified the SAB association with previous events, and the change in the recent paradigm of its genesis.

**Results:**

During the study period, 100 SAB were identified. 85/100 patients were COVID-19 vaccinated. 64% were male and the rest were female. We identified the source of SAB in 90% of cases: in 12% (12/100) it clearly was a previously recent intramuscular vaccine (12/85 vaccinated patients; 14.1% of all COVID-19 vaccinated subjects). All-inclusive, the emergence of SAB was represented as follows: 24% from CRBSI, 23% from skin and soft tissue infections (SSTI), 11% - urinary tract infections (UTI), 10% - respiratory tract infections (RTI), 5% - endovascular reservoirs, 3% from postoperative complications in trauma surgery; septic mouth and bacterial parotiditis were responsible for one case (1% each). Finally, we were not able to identify 10% of the bacteremic fount. Overall, 54 patients died - 54% (46 deaths directly related to the SAB – 85.2%).

**Conclusion:**

SAB always implies an outstanding risk due to its potential complications. Although we previously found an increased incidence of SAB related to COVID-19 vaccines, common causes such as CRBSI and SSTI, are now restored as usual sources. Nevertheless, mortality remains still high amongst our patients.

**Disclosures:**

**All Authors**: No reported disclosures

